# Prognostic value of long-term gamma-glutamyl transferase variability in individuals with diabetes: a nationwide population-based study

**DOI:** 10.1038/s41598-020-72318-7

**Published:** 2020-09-21

**Authors:** Da Young Lee, Kyungdo Han, Ji Hee Yu, Sanghyun Park, Ji A Seo, Nam Hoon Kim, Hye Jin Yoo, Sin Gon Kim, Seon Mee Kim, Kyung Mook Choi, Sei Hyun Baik, Yong Gyu Park, Nan Hee Kim

**Affiliations:** 1 Division of Endocrinology and Metabolism, Department of Internal Medicine, Korea University College of Medicine, Seoul, Republic of Korea; 2 Department of Biostatics, College of Medicine, The Catholic University of Korea, 222, Banpo-daero, Seocho-gu, Seoul, 06591 Republic of Korea; 3 Department of Family Medicine, Korea University College of Medicine, Seoul, Republic of Korea

**Keywords:** Endocrine system and metabolic diseases, Gastroenterology

## Abstract

We examined whether long-term gamma-glutamyl transferase (GGT) variability can predict cardiovascular disease (CVD) and mortality in individuals with diabetes. We included 698,937 Koreans diabetes patients older than 40 years without histories of CVD, chronic liver disease, or heavy alcoholics who received health exams supported by the Korean government more than once in 2009–2012 (baseline). We used Cox proportional analyses to estimate the risk of stroke, myocardial infarction (MI), and all-cause mortality until December 31, 2016, according to the quartiles of the average successive variability (ASV) of GGT measured during the five years before the baseline. A total 26,119, 15,103, and 39,982 cases of stroke, MI, and death, respectively, were found. GGT ASV quartile 4 had a significantly higher risk of stroke and all-cause mortality than quartile 1, with adjustment for risk factors, such as baseline glucose and GGT level, and comorbidities. Hazard ratios (95% confidence intervals) for GGT ASV quartile 4 were 1.06 (1.03–1.10) and 1.23 (1.20–1.27) for stroke and mortality, respectively. This significant association was shown consistently across the baseline GGT quartiles. GGT variability was related to the risk of stroke and all-cause mortality. The effect was most pronounced in all-cause mortality, irrespective of baseline GGT level.

## Introduction

Subjects with diabetes have a two- to three-fold higher mortality rate than the general population^1^. Although management of cardiovascular disease (CVD) promotes the longevity of diabetes patients^2–4^, CVD still constitutes a major cause of death in those with and without diabetes^5,6^.

In addition to the classical risk factors, liver enzymes have shown predictive value in anticipating the development of CVD^7^. Among liver enzymes, gamma-glutamyl transferase (GGT) exhibited a more consistently positive correlation with heart disease, stroke, and short-term all-cause mortality than alanine aminotransferase (ALT) and aspartate aminotransferase (AST)^8,9^. However, the significance of this association was weak in elderly and Asian populations^10,11^. In patients with diabetes, the predictive power of single measurement of GGT was low^12^.

Recently, the intraindividual variability of physiological measures has attracted attention as an emerging risk factor for health-related outcomes. Kim et al. reported that variability in blood glucose and lipid levels, body mass index (BMI), and systolic blood pressure (BP) can predict short-term mortality and cardiovascular events independently, and they showed a graded association between the number of high-variability parameters and cardiovascular outcomes^13^.

Furthermore, the population-wide level of GGT has been reported to be increasing steadily over time in the US and Korea, possibly due to environmental exposure to xenobiotics and increased body iron burden^14^. In addition, considerable intraindividual variability in GGT levels has been reported in the Korean National Health and Nutrition Examination Survey III^15^. Taking into account the close relationship between GGT levels and traditional cardio-metabolic parameters^16^, GGT variability during long-term follow-up might have some correlation with CVD outcomes and longevity.

In the present study, we investigated whether GGT variability can predict the risk of stroke, myocardial infarction (MI), and all-cause mortality during follow up after adjusting for baseline GGT levels in diabetes patients, and we examined whether the prognostic significance of GGT variability is consistent regardless of baseline GGT level in a nationally representative cohort in Korea.

## Methods

### Study design

We used the longitudinal National Health Insurance Sharing Service database. The National Health Insurance System (NHIC) is the single health insurance system managed by the Korean government and includes ~ 97% of the Koreans. NHIC has information including an eligibility database, health examination database, medical bills database submitted by healthcare service providers, and medical care institution database^17,18^. The NHIC recommended to undergo a standardized medical examination every one to two years. The entire database collection is open to researchers.

**Figure 1:**
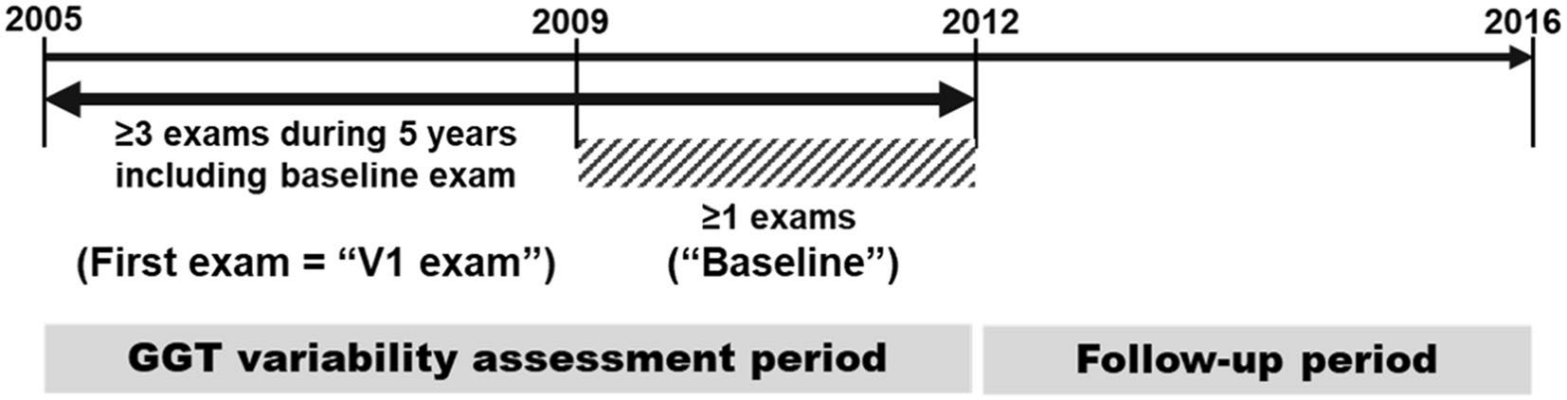
Selection of the study subjects.

As shown in Fig. 1, we chose diabetes patients who had undergone health examinations more than once between January 1, 2009, and December 31, 2012 (“baseline”). Among them, we selected people who also had more than two health examinations during the five years before baseline. The presence of diabetes was defined as the presence of more than one claim per year for the prescription of anti-glycemic agents under the International Classification of Diseases, tenth revision (ICD-10) codes E10–14 or a fasting glucose level ≥ 7 mmol/L.

Among that cohort (Supplementary Figure S1), we excluded those younger than 40 years and those with histories of chronic hepatitis, liver cirrhosis, liver malignancy, stroke, MI, or drinking ≥ 30 g/day of alcohol (estimated by self-reported questionnaires used in the baseline health examination), along with those missing data in the inclusion criteria. After those exclusions, we included 698,937 individuals in this study.

This study protocol was approved by the official review committee of the NHIC and the Institutional Review Board of Korea University Ansan Hospital (Institutional Review Board number 2018AS0161) and was performed in accordance with the Helsinki Declaration of 1975. The requirement of consent has been waived off by the approval of the ethics committee.

### Definition of GGT variability

GGT variability was assessed as the average successive variability (ASV) of serial GGT measurements during the five years before the baseline examination using the following equation:

$$\text{GGT ASV} = \frac{\sum |{x}_{i+1}-{x}_{i}|}{n-1}$$

${x}_{i}$ = each GGT value, $n$ = number of GGT measurements (three to five times per subject).

The GGT level during the baseline examination was also included in the calculation of GGT variability. The first health examination during the five years before baseline is called the “V1 examination” (Fig. 1).

### Study outcomes

Stroke, MI, and death before December 31, 2016, were the end points of this study. The diagnosis of stroke was based on the recording of ICD-10 codes I63 or I64 during an admission record with claims for brain CT or MRI. MI was defined as the recording of ICD-10 codes I21 or I22 with hospitalization. Mortality was identified in nationwide death certificate data from the Korea National Statistical Office. The time between the last examination in 2009–2012 and the development of outcomes or December 31, 2016, is defined as the follow-up period.

### Anthropometric and laboratory measurements

The study subjects completed a structured questionnaire about their demographic information, lifestyle behaviors, medical history, and medication use during their medical examinations. Smoking history was categorized as never, ex-, and current smokers. Alcohol drinking was divided into near abstinence or moderate (< 30 g/day). Regular exercise was defined > 20 min of vigorous- or > 30 min of moderate-intensity exercise at least once per week^19^.

Waist circumference was checked at the middle point between the rib cage and the iliac crest. BP was measured using a standardized sphygmomanometer after 5 min of rest. The presence of hypertension was defined as a systolic BP ≥ 140 mmHg, diastolic BP ≥ 90 mmHg, or the presence of more than one claim per year for the prescription of antihypertensive medications under ICD-10 codes I10–I15. Income status was dichotomized at the lowest 20%.

After an overnight fast of at least 8 h, venous blood sampling was done in the morning. Serum levels of AST, ALT, GGT, creatinine, hemoglobin, fasting glucose, total cholesterol, triglycerides, high-density lipoprotein cholesterol, and low-density lipoprotein cholesterol (LDL-C) were measured. Quality control for the laboratory tests was conducted in accordance with the Korean Association of Laboratory Quality Control. We classified the estimated glomerular filtration rate (eGFR) < 60 mL/minute/1.73 m^2^ calculated by the Modification of Diet in Renal Disease formula^20^ as chronic kidney disease (CKD)^21^. Dyslipidemia was determined as total cholesterol levels ≥ 6**.**21 mmol/L or the presence of more than one claim per year for the prescription of anti-hyperlipidemic agents under ICD-10 code E78.

Diabetic retinopathy was defined as the presence of ICD-10 codes H36.0, and peripheral artery disease (PAD) was identified by the presence of two or more ICD-10 codes (I70 and I73) in the outpatient setting and one or more ICD-10 codes (I70 and I73) in the inpatient setting^22^. The number of prescriptions for anti-diabetic medication during the 12 months preceding baseline was identified. Admission episodes for heart failure were identified using claims data for hospital admission.

### Statistical analysis

We stratified study subjects according to quartiles of baseline GGT and GGT ASV. The baseline characteristics were compared by quartile using chi-square tests for categorical variables and an analysis of variance for continuous variables. AST, ALT, GGT, and triglyceride levels were log-transformed for analysis. Data are presented as number (%), means ± standard deviations (SD), or geometric means (95% confidence intervals [CIs]).

To assess the risk of stroke, MI, and all-cause mortality during follow up, we performed Cox proportional hazard analyses by the quartiles of baseline GGT and GGT ASV, using each quartile 1 as the reference. We adjusted for confounders at baseline using two models. Model 1 was adjusted for age, sex, eGFR, BMI, moderate drinking, current smoking, regular exercise, presence of hypertension or dyslipidemia, hemoglobin, and lowest 20% income. Model 2 is the same as model 1 plus adjustments for glucose, duration of diabetes, the number of prescriptions for oral anti-diabetic medication, prescriptions for insulin, and the presence of diabetic retinopathy and peripheral artery disease. In the GGT variability analysis, the baseline GGT level was also included as a confounding factor. In addition, we examined the effects of GGT variability on incident stroke, MI, and all-cause mortality according to the baseline GGT quartiles to show that our results are consistent regardless of GGT level.

We performed subgroup analyses by dividing the subjects by age; sex; obesity; eGFR; anemia; current smoking; alcohol drinking; income status; the presence of hypertension, dyslipidemia, diabetic retinopathy, or PAD; duration of diabetes; number of prescriptions for oral anti-diabetic medication; and prescriptions for insulin. In these analyses, we obtained hazard ratios (HRs) and 95% CIs for stroke, MI, and all-cause mortality in GGT ASV quartile 4 versus quartiles 1–3 using model 2 except for the variable that categorized each subgroup.

For the Cox proportional hazards analyses, we found a variable inflation factor for all covariables of less than 2.0, and we found no relevant multicollinearity among covariates. SAS version 9.3 (SAS Institute Inc., Cary, NC, USA) was used for statistical analyses. A *p* value of < 0.05 was considered to be statistically significant.

## Results

Subjects in GGT ASV quartile 4 were more obese, had higher BP, and were more likely to be current smokers, moderate drinkers, and have hypertension, dyslipidemia, CKD, or PAD than subjects in quartile 1, despite having a shorter duration of diabetes (Table 1). On the other hand, subjects in the lowest quartile for baseline GGT level were older and had a higher prevalence of CKD and PAD, but they had a lower prevalence of hypertension and obesity than the other quartiles (Supplementary Table S1).

**Table 1:** Baseline characteristics of the study subjects by quartiles of gamma-glutamyl transferase variability assessed using average successive variability^a^.

Characteristics	ASV Q1 (n = 174,767)	ASV Q2 (n = 174,658)	ASV Q3 (n = 174,791)	ASV Q4 (n = 174,721)	*P* value
Age (years)	58.5 ± 12.0	58.1 ± 11.9	58.0 ± 11.9	58.5 ± 11.7	< 0.001
Sex, male (%)	103,211 (59.1)	103,172 (59.1)	103,252 (59.1)	103,211(59.1)	0.999
BMI (kg/m^2^)	24.8 ± 3.2	25.0 ± 3.2	25.1 ± 3.2	25.0 ± 3.3	< 0.001
WC (cm)	84.7 ± 8.4	85.0 ± 8.3	85.3 ± 8.3	85.4 ± 8.3	< 0.001
Systolic BP (mmHg)	128.2 ± 15.2	128.4 ± 15.2	128.5 ± 15.2	128.6 ± 15.4	< 0.001
Fasting glucose (mg/dL)	143.2 ± 41.9	142.9 ± 42.2	142.1 ± 42.8	140.8 ± 43.5	< 0.001
Triglycerides (mg/dL)	137.1 (136.8–137.5)	142.0 (141.6–142.3)	145.9 (145.5–146.3)	148.8 (148.4–149.2)	< 0.001
HDL-C (mg/dL)	51.2 ± 17.9	51.2 ± 18.8	51.2 ± 18.9	51.6 ± 18.3	< 0.001
LDL-C (mg/dL)	113.3 ± 43.2	112.8 ± 43.6	111.4 ± 42.6	109.4 ± 43.8	< 0.001
AST (U/L)	24.7 (24.6–24.7)	25.4 (25.3–25.4)	26.3 (26.2–26.3)	27.9 (27.9–28.0)	< 0.001
ALT (U/L)	24.7 (24.6–24.7)	25.9 (25.8–25.9)	27.0 (26.9–27.1)	28.5 (28.5–28.6)	< 0.001
GGT (U/L)	29.2 (29.1–29.27)	31.6 (31.5–31.7)	34.5 (34.4–34.6)	41.2 (41.1–41.4)	< 0.001
V1 GGT	29.4 (29.3–29.5)	32.2 (32.1–32.3)	35.7 (35.6–35.8)	43.5 (43.3–43.6)	< 0.001
GGT ASV	1.1 (1.1–1.1)	1.2 (1.2–1.2)	1.4 (1.4–1.4)	1.9 (1.9–1.9)	< 0.001
Hemoglobin (g/dL)	14.0 ± 1.6	14.0 ± 1.6	14.0 ± 1.6	13.9 ± 1.6	< 0.001
Current smoker (%)	37,153 (21.3)	38,578 (22.1)	39,234 (22.5)	39,462 (22.6)	< 0.001
Moderate drinking (%)	64,407 (36.9)	66,921 (38.3)	68,836 (39.4)	70,144 (40.2)	< 0.001
Regular exercise (%)	40,916 (23.4)	40,456 (23.2)	39,882 (22.8)	39,317 (22.5)	< 0.001
Duration of diabetes (years)	3.5 ± 3.5	3.5 ± 3.4	3.4 ± 3.4	3.39 ± 3.3	< 0.001
≥ 5 years (%)	66,916 (38.3)	64,063 (36.7)	62,044 (35.5)	59,459 (34.0)	
Diabetic retinopathy (%)	27,900 (16.0)	26,963 (15.4)	26,819 (15.34)	27,374 (15.7)	< 0.001
**Comorbidities**					
Hypertension (%)	94,976 (54.3)	96,558 (55.3)	99,170 (56.7)	103,254 (59.1)	< 0.001
Dyslipidemia (%)	68,646 (39.3)	72,098 (41.3)	75,409 (43.1)	80,466 (46.1)	< 0.001
CKD (%)	19,577 (11.2)	19,703 (11.3)	20,183 (11.6)	20,920 (12.0)	< 0.001
PAD (%)	42,129 (24.1)	42,512 (24.3)	44,007 (25.2)	47,282 (27.1)	< 0.001
Admission for HF (%)	501 (0.3)	526 (0.3)	613 (0.4)	869 (0.5)	< 0.001
Income (lowest 20%, %)	36,528 (20.9)	36,531 (20.9)	36,376 (20.8)	37,578 (21.5)	< 0.001
**Year of V1 exam (%)**					< 0.001
2005	51,278 (29.3)	57,626 (33.0)	56,368 (32.3)	50,891 (29.1)	
2006	56,978 (32.6)	56,186 (32.2)	56,952 (32.6)	58,653 (33.6)	
2007	27,087 (15.5)	25,366 (14.5)	25,646 (14.7)	27,735 (15.9)	
2008	25,635 (14.7)	23,872 (13.7)	24,717 (14.1)	26,511 (15.2)	
2009	10,434 (6.0)	8,866 (5.1)	8,566 (4.9)	8,660 (5.0)	
2010	3,355 (1.9)	2,742 (1.6)	2,542 (1.5)	2,271 (1.3)	
GGT variability assessment period (years)	3.6 ± 0.7	3.7 ± 0.6	3.7 ± 0.6	3.7 ± 0.6	< 0.001

^a^Q1: 1–1.19 (men), 1–1.17 (women) U/L; Q2: 1.19–1.32 (men), 1.17–1.31 (women) U/L; Q3: 1.32–1.53 (men), 1.31–1.52 (women) U/L; Q4: 1.53–54.21 (men), 1.52–58.65 (women) U/L.

Data are presented as means ± standard deviations, geometric means (95% confidence intervals), or numbers (%). One-way analysis of variance and chi-squared testing were used to compare the characteristics of the study subjects at baseline. Post-hoc multiple comparison analyses were performed with Bonferroni correction. AST, ALT, GGT, and triglyceride levels were log-transformed for analysis.

ALT, alanine aminotransferase; AST, aspartate transaminase; ASV, average successive variability; BMI, body mass index; BP, blood pressure; CKD, chronic kidney disease; GGT, gamma-glutamyl transpeptidase; HDL-C, high-density lipoprotein-cholesterol; HF, heart failure; LDL-C, low-density lipoprotein-cholesterol; PAD, peripheral artery disease; WC, waist circumference.

**Figure 2:**
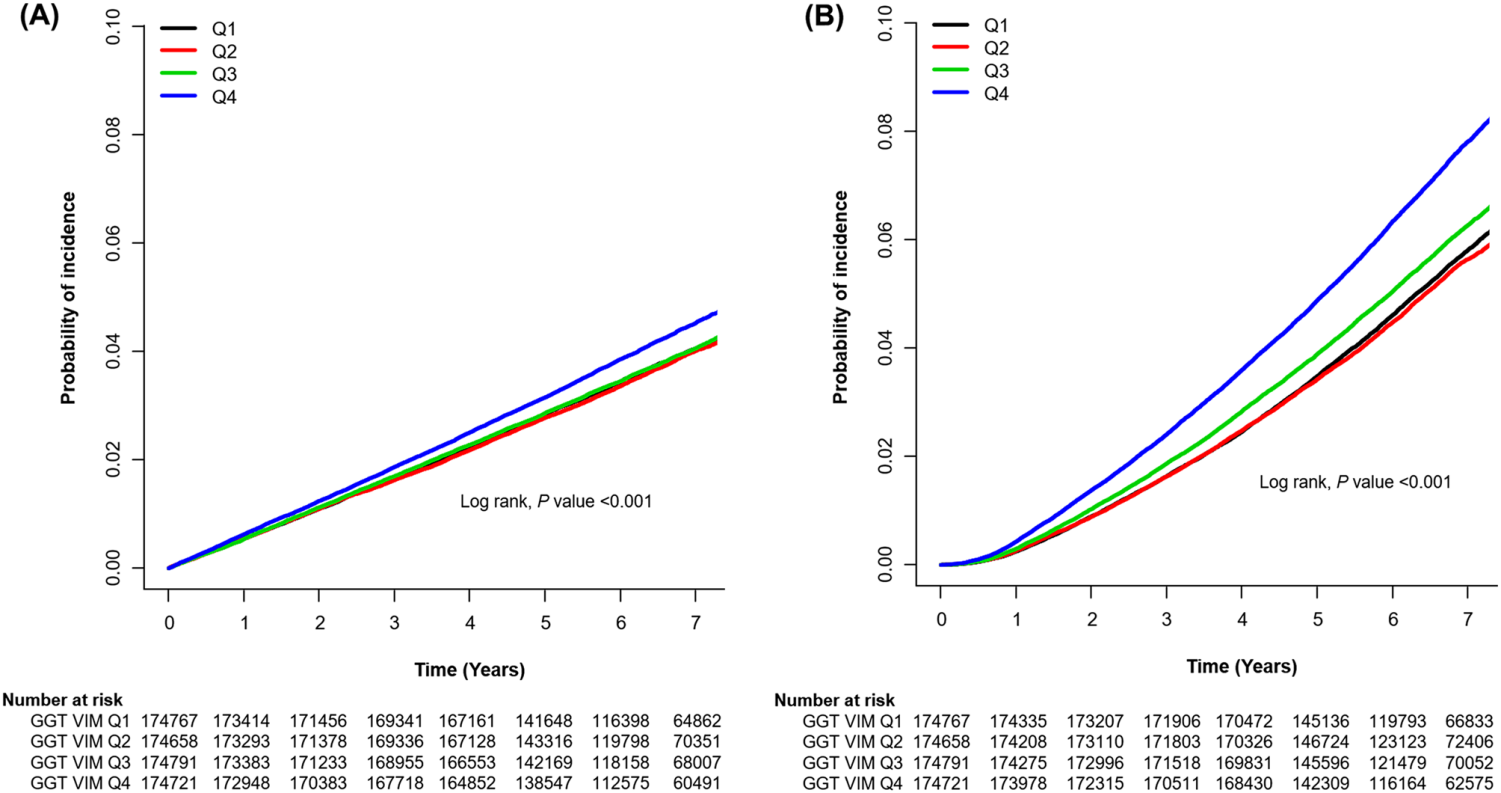
Kaplan–Meier curves for the incidence of stroke and all-cause mortality according to quartiles of gamma-glutamyl transpeptidase variability assessed by average successive variability. (**A**) Stroke. (**B**) All-cause mortality.

As shown in Table 2 and Fig. 2, subjects in GGT ASV quartile 4 had highest incidence rate and HR for stroke, MI, and all-cause mortality during follow up, though the statistical significance for MI was weakened in the fully adjusted model. Subjects in GGT ASV quartile 4 had a 6% higher risk for stroke and a 23% higher risk for all-cause mortality during follow up compared with those in GGT ASV quartile 1, even after adjustment for baseline GGT.

**Table 2:** Hazard ratios and 95% confidence intervals for the incidence of stroke, myocardial infarction, and all-cause mortality during follow up by quartiles of average successive variability of gamma-glutamyl transpeptidase ^a^.

	Events (n)	Follow-up duration (person-years)	Incidence rate (per 1,000 person-years)	Unadjusted HR (95% CI)	Multivariate-adjusted HR (95% CI)	
					Model 1	Model 2
**Stroke**						
Q1 (n = 174,767)	6,366	1,080,230.2	5.89	1(Ref.)	1(Ref.)	1(Ref.)
Q2 (n = 174,658)	6,312	1,090,190.4	5.79	0.98 (0.94‒1.02)	1.01 (0.98‒1.05)	1.01 (0.98‒1.05)
Q3 (n = 174,791)	6,430	1,084,103.9	5.93	1.01 (0.97‒1.04)	1.03 (0.99‒1.06)	1.03 (0.99‒1.06)
Q4 (n = 174,721)	7,011	1,063,802.0	6.59	1.12 (1.08‒1.16)	1.07 (1.04‒1.11)	1.06 (1.03‒1.10)
**Myocardial infarction**						
Q1 (n = 174,767)	3,673	1,088,178.6	3.38	1(Ref.)	1(Ref.)	1(Ref.)
Q2 (n = 174,658)	3,763	1,097,406.4	3.43	1.01 (0.97‒1.06)	1.04 (0.99‒1.09)	1.04 (0.99‒1.09)
Q3 (n = 174,791)	3,842	1,091,525.6	3.52	1.04 (0.99‒1.09)	1.06 (1.01‒1.11)	1.06 (1.01‒1.11)
Q4 (n = 174,721)	3,825	1,072,766.6	3.57	1.06 (1.01‒1.11)	1.03 (0.98‒1.07)	1.02 (0.97‒1.07)
**All-cause mortality**						
Q1 (n = 174,767)	9,037	1,097,781.3	8.23	1(Ref.)	1(Ref.)	1(Ref.)
Q2 (n = 174,658)	8,887	1,107,422.2	8.02	0.97 (0.94‒1.00)	1.02 (0.99‒1.05)	1.02 (0.99‒1.05)
Q3 (n = 174,791)	9,891	1,101,602.7	8.98	1.09 (1.06‒1.12)	1.12 (1.09‒1.16)	1.12 (1.09‒1.16)
Q4 (n = 174,721)	12,167	1,082,782.3	11.24	1.37 (1.33‒1.41)	1.24 (1.20‒1.28)	1.23 (1.20‒1.27)

^a^Q1: 1–1.19 (men), 1–1.17 (women) U/L; Q2: 1.19–1.32 (men), 1.17–1.31 (women) U/L; Q3: 1.32–1.53 (men), 1.31–1.52 (women) U/L; Q4: 1.53–54.21 (men), 1.52–58.65 (women) U/L.

Model 1 is adjusted for age, sex, baseline estimated glomerular filtration rate, body mass index, moderate drinking, current smoking, regular exercise, presence of hypertension and dyslipidemia, hemoglobin, lowest 20% income, and baseline GGT. Model 2 is the same as model 1, plus adjustment for glucose, duration of diabetes, number of prescriptions for oral anti-diabetic medication, prescriptions for insulin, and presence of diabetic retinopathy and peripheral artery disease.

Meanwhile, in the baseline GGT quartiles, the incidence rate and risk of stroke, MI, and all-cause mortality in the crude model were highest in baseline GGT quartile 1 (Supplementary Table S2). After adjustment for several confounders, including age, the risk of all three outcomes was the highest in quartile 4.

**Figure 3:**
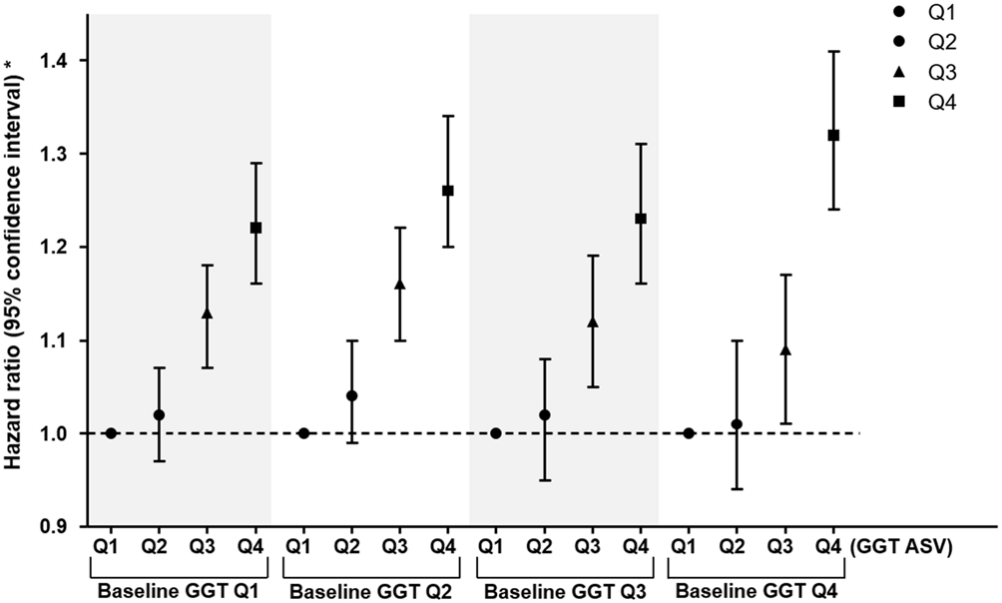
Risk of all-cause mortality during follow up after dividing subjects by quartiles of gamma-glutamyl transpeptidase variability assessed by average successive variability in each quartile of baseline gamma-glutamyl transpeptidase. GGT, gamma-glutamyl transpeptidase. * Adjusted for age, sex, baseline estimated glomerular filtration rate, body mass index, moderate drinking, current smoking, regular exercise, presence of hypertension and dyslipidemia, hemoglobin, lowest 20% income, glucose, duration of diabetes, number of prescriptions for oral anti-diabetic medication, prescriptions for insulin, and presence of diabetic retinopathy and peripheral artery disease.

To evaluate whether the effect of GGT ASV was consistent irrespective of the GGT value, we further divided the subjects by both GGT ASV and baseline GGT quartiles. The significance in all-cause mortality during follow up was maintained across the baseline GGT quartiles (Fig. 3 and Supplementary Table S3). However, the increased risk for stroke in GGT ASV quartile 4 was found only in subjects in baseline GGT quartiles 1 and 3 (Supplementary Table S3).

**Figure 4:**
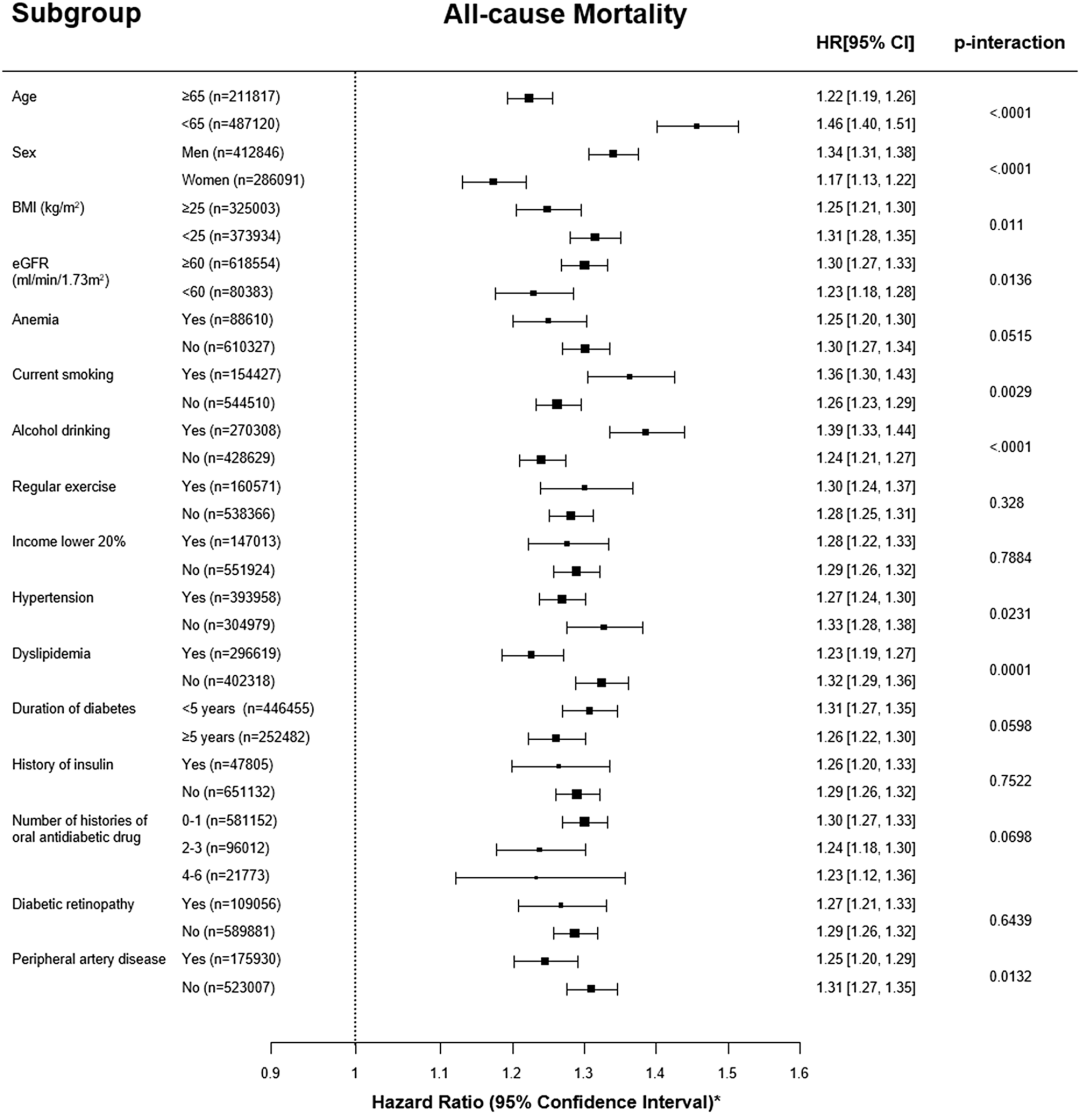
Hazard ratios and 95% confidence intervals for the incidence of all-cause mortality during follow up in the highest quartile versus the other three quartiles of gamma-glutamyl transferase variability assessed by average successive variability in subgroup analyses**.** * Adjusted for age, sex, baseline estimated glomerular filtration rate, body mass index, moderate drinking, current smoking, regular exercise, presence of hypertension and dyslipidemia, hemoglobin, lowest 20% income, glucose, duration of diabetes, number of prescriptions for oral anti-diabetic medication, prescriptions for insulin, and presence of diabetic retinopathy and peripheral artery disease.

In the subgroup analyses, subjects in GGT ASV quartile 4 consistently had an increased risk for all-cause mortality and stroke during follow up, with the exception of those with a history of insulin treatment or 4–6 classes of oral antidiabetic drugs in the stroke analysis (Fig. 4 and Supplementary Figure S2). However, the subgroup analyses for MI showed no significance (Supplementary Figure S3).

## Discussion

In this nationwide population-based study in diabetes patients, we demonstrated that GGT variability is an independent indicator of stroke and death during follow up, irrespective of baseline GGT levels. We also confirmed that the GGT level is associated with the development of CVD and death.

Previous studies about CVD or short-term mortality in individuals with diabetes yielded limited significance^12,23–26^. Ndrepepa et al. showed that a high GGT level was independently associated with the risk of short-term all-cause mortality in patients with diabetes and coronary artery disease^26^. However, they enrolled only 1,448 patients with diabetes, and their observation period was only three years. In a pooled analysis of three British health surveys, higher GGT levels were a risk factor for CVD-related mortality, but GGT did not improve CVD risk prediction compared with traditional risk factors^24^. Two observational cohort studies conducted in Europe also included fewer than 2000 diabetes patients^23,27^. Regarding CVD risk in diabetes patients, the predictive value of the GGT level was weak^12,25^. Furthermore, the cutoff value defining elevated GGT varied from study to study^23,24,26,27^. The different predictive powers found in different studies might be partially attributable to those various cutoff points. Unlike previous studies, our data confirm that the GGT level has strong predictive value for stroke, MI, and all-cause mortality during follow up in diabetes patients (Supplementary Table S2).

Several mechanisms have been proposed to explain the association between GGT and cardiometabolic parameters and mortality, independently of alcohol per se^16^. (1) Several studies showed that GGT not only reflects hepatitis and non-alcoholic fatty liver disease^28^, but also correlates with visceral fat accumulation, hepatic triglyceride deposits, metabolic syndrome, and type 2 diabetes^29,30^, which are themselves risk factors for CVD and premature death^31,32^. These features were also found in our study. As shown in Supplementary Table S1, individuals of GGT Q4 exhibited higher BMI and BP, and poorer lipid profiles compared with GGT Q1, despite younger age. (2) As a key enzyme in glutathione metabolism, which is a human antioxidant^33^, GGT is a marker of oxidative stress^28^. (3) GGT can promote LDL-C oxidation, which enhances vulnerability to plaques, platelet aggregation, and thrombosis^34^. Given that both diabetes and elevated GGT are conditions associated with heightened inflammatory burden and activated plaque progression and instability^35,36^, it is plausible that they might act synergistically in increasing the risk of CVD.

The meaning of increased GGT variability is not yet clear. Several recent publications have considered the role of fluctuations in blood glucose, blood pressure, lipid, and body weight on the development of diabetes, CVD, and early death regardless of baseline variables^13,37^. Some basic research data indicate that fluctuations in blood glucose and blood pressure can increase oxidative stress more than continuous hyperglycemia or hypertension^38,39^. Although we do not know the exact mechanism by which GGT variability affects stroke and short-term all-cause mortality in diabetes patients, it might be a reflection of changing oxidative stress, which can damage cells. In addition, GGT variability might induce the vulnerable plaques similar to LDL-C variability^40^.

Of note, the incidence rate and unadjusted HRs for CVD and death during the follow-up period were rather higher in the subjects in baseline GGT quartile 1 than in those in quartiles 3 and 4 (Supplementary Table S2), and after adjustment for several factors, a positive association was shown. This could be because the subjects in baseline GGT quartile 1 were older, slender, and more likely to have PAD or CKD than those in the other quartiles. On the other hand, increased GGT variability consistently showed a higher incidence rate and HR in all models and subgroup analyses. Therefore, GGT variability could reinforce the predictive value of single-measurement GGT for stroke and all-cause mortality.

The statistical significance of GGT variability was weak in the CVD outcomes. Statins could be one factor responsible for modifying that relationship. Nearly half of our study subjects had dyslipidemia, and most of that half were expected to take a statin or had a history of statin use^41^. Statin therapy is associated with a reduced risk of CVD events and early mortality^42^. We also found that patients with dyslipidemia had lower mortality and stroke incidence during the follow-up period than those without dyslipidemia, which again reflects the beneficial effects of statin use. Some experimental evidence suggests that statins alter liver enzymes and stabilize atherosclerotic plaques^43^ and suppress the expression of GGT in aortic atherosclerotic plaques^44^.

In the present study, we showed that GGT variability correlated more strongly with death during the follow-up period than with CVD. Because we diagnosed incident stroke and MI using only ICD-10 codes and admission history, we might have underdiagnosed them. Additionally, deaths caused by undiagnosed CVD are possible. Future studies should clarify the causes of death to explain this finding. Little significance in MI might be associated with lower incidence rate compared with stroke and death.

We also found that young, male individuals who currently smoke and consume high amounts of alcohol exhibited a remarkably high follow-up mortality risk according to GGT variability (Fig. 4). The function of glutathione seems to decline with age^45^, smoking, and alcohol drinking^33,46^, which could explain that result. Serum GGT levels increase proportionally with the amount of glutathione conjugates, which indicates an ability to clear oxidative stress, rather than the amount of oxidative stress itself. Therefore, because reactive production of glutathione conjugates in response to external stimulation is impaired in the elderly, serum GGT levels in the elderly might not reflect the extent of oxidative stress as well as they do in younger people^45^. Additionally, other cardiometabolic variables, such as BMI, hypertension, and dyslipidemia, interacted significantly with the association between GGT variability and all-cause mortality, which was in line with a prior general population–based study from the NHISS database that used a single GGT measurement^47^. Therefore, we need to identify possible contributors to increased GGT variability in relatively young individuals with diabetes to reduce their risk of an early death.

We are aware of some limitations of the present study. First, the inclusion criteria for this study, which were based on the number of health check-ups, could be a bias because men and employees tend to participate in health check-ups more regularly than others^48^. Second, we could not assess the exact cause of death. Third, given that this is an observational study, we cannot confirm causality in the relationship between GGT variability and the study outcomes. To overcome the possibility of reverse causality, we conducted various subgroup analyses, and they showed consistent significance. Finally, we did not consider changes in factors that could affect GGT variability and related cardiovascular risk factors, such as diet and exercise during the follow-up period. Nevertheless, this is the first study to investigate the significance of GGT variability in predicting CVD and all-cause mortality risk during follow up in a nationally representative cohort of diabetes patients. We were able to include nearly all patients with diabetes in Korea and adjust for several essential risk factors, such as duration of diabetes, anti-diabetic medication, and diabetes-related complications.

In conclusion, GGT variability is associated with a significantly increased risk of all-cause mortality and stroke during the follow-up period regardless of baseline GGT levels, and the correlation is stronger with all-cause mortality. Therefore, it is important to identify the factors that contribute to increased GGT variability to extend the lives of patients with diabetes.

## Supplementary information

Supplementary information.

## Data Availability

The data that support the findings of this study are available from the National Health Insurance Corporation but restrictions apply to the availability of these data, which were used under license for the current study, and so are not publicly available. Data are however available from the authors upon reasonable request and with permission of the National Health Insurance Corporation.

## Abbreviations

CVD: Cardiovascular disease
GGT: Gamma-glutamyl transferase
ALT: Alanine aminotransferase
AST: Aspartate aminotransferase
BMI: Body mass index
BP: Blood pressure
MI: Myocardial infarction
NHIC: National Health Insurance System
ICD-10: International Classification of Diseases, tenth revision
ASV: Average successive variability
LDL-C: Low-density lipoprotein cholesterol
eGFR: Estimated glomerular filtration rate
CKD: Chronic kidney disease
PAD: Peripheral artery disease
SD: Standard deviation
CI: Confidence interval
HR: Hazard ratio
