# Supplementary material for: Prognostic value of long-term gamma-glutamyl transferase variability in individuals with diabetes: a nationwide population-based study
Source: Sci Rep. 2020 Sep 21;10:15375. doi: 10.1038/s41598-020-72318-7 (PMC7506020; doi:10.1038/s41598-020-72318-7)
Supplement: Supplementary file 1 — Supplementary information. [file 41598_2020_72318_MOESM1_ESM.docx]

**Prognostic value of long-term gamma-glutamyl transferase variability in individuals with diabetes: a nationwide population-based study**

**Running Title**: Gamma-glutamyl Transferase Variability and Mortality in Individuals with Diabetes

**Da Young Lee^1^, Kyungdo Han^2^, Ji Hee Yu^1^, Sanghyun Park^2^, Ji A Seo^1^, Nam Hoon Kim^1^, Hye Jin Yoo^1^, Sin Gon Kim^1^, Seon Mee Kim^3^, Kyung Mook Choi^1^, Sei Hyun Baik ^1^, Yong Gyu Park^2^ & Nan Hee Kim^1^**

^1^Division of Endocrinology and Metabolism, Department of Internal Medicine, Korea University College of Medicine, Seoul, Republic of Korea. ^2^Department of Biostatics, College of Medicine, The Catholic University of Korea, Seoul, Republic of Korea. ^3^Department of Family Medicine, Korea University College of Medicine, Seoul, Republic of Korea.

Da Young Lee and Kyungdo Han contributed equally to this article.

Correspondence and requests for materials should be addressed to N.H.K. (email: nhkendo@gmail.com) or Y.G.P. (email: ygpark@catholic.ac.kr)

**Supplementary Information**

**Supplementary Figure S1.** Flow chart of the study population**.**

**Supplementary Table S1.** Baseline characteristics of the study subjects by quartiles of baseline gamma-glutamyl transferase.

**Supplementary Table S2.** Hazard ratios and 95% confidence intervals for the incidence of stroke, myocardial infarction, and all-cause mortality during follow up by quartiles of baseline gamma-glutamyl transferase.

**Supplementary Table S3.** Hazard ratios and 95% confidence intervals for the incidence of stroke, myocardial infarction, and all-cause mortality during follow up by quartiles of gamma-glutamyl transpeptidase variability assessed by average successive variability in each quartile of baseline gamma-glutamyl transpeptidase.

**Supplementary Figure S2.** Hazard ratios and 95% confidence intervals for the incidence of stroke in the highest quartile versus the other three quartiles of gamma-glutamyl transferase variability assessed by average successive variability in subgroup analyses adjusted for age, sex, baseline estimated glomerular filtration rate, body mass index, moderate drinking, current smoking, regular exercise, presence of hypertension and dyslipidemia, hemoglobin, lowest 20% income, glucose, duration of diabetes, number of prescriptions for oral anti-diabetic medication, prescriptions for insulin, and presence of diabetic retinopathy and peripheral artery disease.

**Supplementary Figure S3.** Hazard ratios and 95% confidence intervals for the incidence of myocardial infarction in the highest quartile versus the other three quartiles of gamma-glutamyl transferase variability assessed by average successive variability in subgroup analyses adjusted for age, sex, baseline estimated glomerular filtration rate, body mass index, moderate drinking, current smoking, regular exercise, presence of hypertension and dyslipidemia, hemoglobin, lowest 20% income, glucose, duration of diabetes, number of prescriptions for oral anti-diabetic medication, prescriptions for insulin, and presence of diabetic retinopathy and peripheral artery disease.


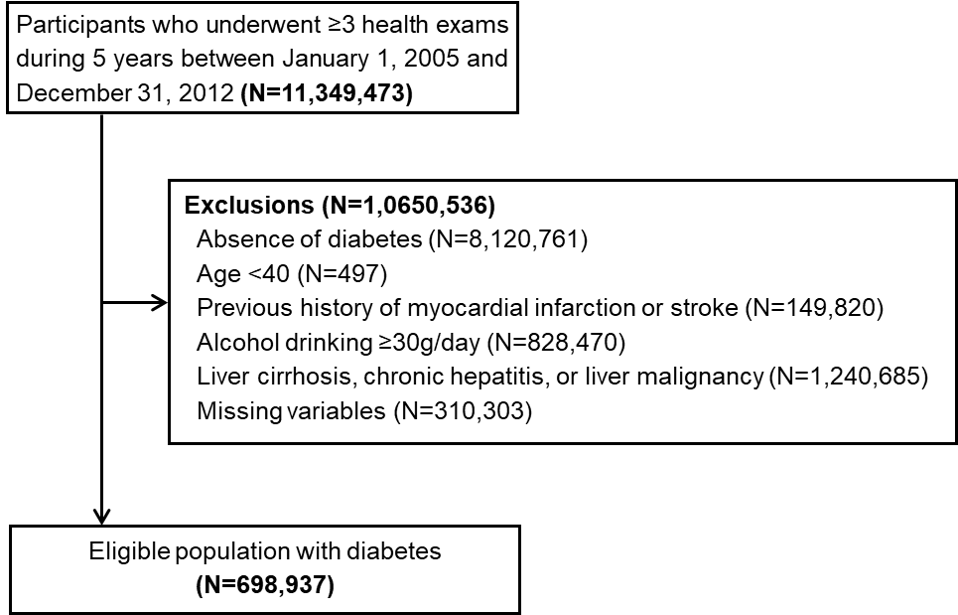


**Supplementary Figure S1. Flow chart of the study population**

| **Supplementary Table S1. Baseline characteristics of the study subjects by quartiles of baseline gamma-glutamyl transferase ^a^** | | | | | |
| --- | --- | --- | --- | --- | --- |
| **Characteristics** | **GGT Q1**  **(n =171673)** | **GGT Q2**  **(n =** **185682)** | **GGT Q3**  **(n =** **168109)** | **GGT Q4**  **(n =** **173473)** | ***P* value** |
| Age (years) | 60.4 ± 12.4 | 59.5 ± 11.5 | 57.6 ± 11.5 | 55.6 ± 11.5 | <0.001 |
| Sex, male (%) | 106009 (61.8) | 103428 (55.7) | 101435 (60.3) | 101974 (58.8) | <0.001 |
| BMI (kg/m^2^) | 23.7 ± 2.9 | 24.7 ± 3.0 | 25.5 ± 3.1 | 26.0 ± 3.4 | <0.001 |
| WC (cm) | 82.3 ± 8.1 | 84.5 ± 8.1 | 86.3 ± 8.1 | 87.2 ± 8.3 | <0.001 |
| Systolic BP (mmHg) | 126.0 ± 15.2 | 127.9 ± 15.1 | 129.2 ± 15.0 | 130.6 ± 15.3 | <0.001 |
| Fasting glucose (mg/dL) | 135.1 ± 40.5 | 139.8 ± 42.0 | 144.5 ± 42.9 | 149.7 ± 43.6 | <0.001 |
| Total cholesterol (mg/dL) | 182.8 ± 36.8 | 193.1 ± 38.8 | 199.5 ± 40.2 | 206.1 ± 43.4 | <0.001 |
| Triglycerides (mg/dL) | 111.4 (111.2-111.7) | 134.3 (134.0-134.6) | 156.2 (155.9-156.6) | 181.6 (181.1-182.1) | <0.001 |
| HDL-C (mg/dL) | 51.0 ± 18.6 | 51.0 ± 19.3 | 50.8 ± 18.1 | 52.2 ± 17.6 | <0.001 |
| LDL-C (mg/dL) | 107.5 ± 40.1 | 112.7 ± 42.8 | 114.1 ± 44.0 | 112.6 ± 45.9 | <0.001 |
| AST (U/L) | 21.9 (21.9-21.9) | 23.6 (23.5-23.6) | 26.4 (26.3-26.4) | 33.9 (33.8-34.0) | <0.001 |
| ALT (U/L) | 19.4 (19.4-19.4) | 23.2 (23.2-23.3) | 28.5 (28.4-28.5) | 38.6 (38.5-38.7) | <0.001 |
| GGT (U/L) | 16.5 (16.4-16.5) | 25.5 (25.5-25.6) | 38.9 (38.8-38.9) | 81.6 (81.4-81.9) | <0.001 |
| V1 GGT (U/L) | 21.4 (21.3-21.4) | 28.5 (28.5-28.6) | 39.1 (39.0-39.2) | 62.5 (62.3-62.7) | <0.001 |
| GGT ASV | 1.4 (1.4-1.4) | 1.3 (1.3-1.3) | 1.4 (1.4-1.4) | 1.5 (1.5-1.5) | <0.001 |
| Hemoglobin (g/dL) | 13.6 ± 1.5 | 13.8 ± 1.5 | 14.2 ± 1.5 | 14.3 ± 1.6 | <0.001 |
| Current smoker (%) | 29790 (17.4) | 35368(19.1) | 40600 (24.2) | 48669 (28.1) | <0.001 |
| Moderate drinking (%) | 48527 (28.3) | 61824 (33.3) | 71752 (42.7) | 88205 (50.9) | <0.001 |
| Regular exercise (%) | 44842 (26.1) | 44088 (23.7) | 36883 (21.9) | 34758 (20.0) | <0.001 |
| Duration of diabetes (years) | 4.3 ± 3.5 | 3.7 ± 3.4 | 3.2 ± 3.3 | 2.6 ± 3.1 | <0.001 |
| ≥5 years (%) | 80027 (46.6) | 73726 (39.7) | 54910 (32.7) | 43819 (25.3) |  |
| Diabetic retinopathy (%) | 37106 (21.6) | 32323 (17.4) | 22220 (13.2) | 17407 (10.0) | <0.001 |
| Comorbidities |  |  |  |  |  |
| Hypertension (%) | 88150 (51.4) | 103916 (56.0) | 97707 (58.1) | 104185 (60.1) | <0.001 |
| Dyslipidemia (%) | 59859 (34.9) | 76967 (41.5) | 75304 (44.8) | 84489 (48.7) | <0.001 |
| CKD (%) | 22186 (12.9) | 22682 (12.2) | 18666 (11.1) | 16849 (9.7) | <0.001 |
| PAD (%) | 46710 (27.2) | 49763 (26.8) | 41272 (24.6) | 38185 (22.0) | <0.001 |
| Admission for HF (%) | 606 (0.4) | 677 (0.4) | 571 (0.3) | 655 (0.4) | 0.288 |
| Income (lower 20%, %) | 35324 (20.6) | 39409 (21.2) | 35244 (21.0) | 37036 (21.4) | <0.001 |
| Year of V1 exam (%) |  |  |  |  | <0.001 |
| 2005 | 50571 (29.5) | 56097 (30.2) | 53443 (31.8) | 56052 (32.3) |  |
| 2006 | 57373 (33.4) | 61997 (33.4) | 54976 (32.7) | 54423 (31.4) |  |
| 2007 | 26571 (15.5) | 28643 (15.4) | 24776 (14.7) | 25844 (14.9) |  |
| 2008 | 25526 (14.9) | 26824 (14.5) | 23659 (14.1) | 24726 (14.3) |  |
| 2009 | 9040 (5.3) | 9477 (5.1) | 8601 (5.1) | 9408 (5.4) |  |
| 2010 | 2592 (1.5) | 2644 (1.4) | 2654 (1.6) | 3020 (1.7) |  |
| GGT variability  assessment period (years) | 3.7 ± 0.6 | 3.7 ± 0.6 | 3.7 ± 0.6 | 3.6 ± 0.7 | <0.001 |
| ^a^ Q1: 4–25 (men), 4–16 (women) U/L; Q2: 26–38 (men), 17–23 (women) U/L; Q3: 39– 63 (men), 24–33 (women) U/L; Q4: 64–1000 (men), 34– 1000 (women) U/L.  Data are presented as means ± standard deviations, geometric means (95% confidence intervals), or numbers (%). One-way analysis of variance and chi-squared testing were used to compare the characteristics of the study participants at baseline. Post-hoc multiple comparison analyses were performed with Bonferroni correction. AST, ALT, GGT, and triglyceride levels were log-transformed for analysis.  Abbreviations: ASV, average successive variability; BMI, body mass index; WC, waist circumference; BP, blood pressure; TC, total cholesterol; HDL-C, high-density lipoprotein-cholesterol; LDL-C, low-density lipoprotein-cholesterol; AST, aspartate transaminase; ALT, alanine aminotransferase; GGT, gamma-glutamyl transpeptidase; CKD, chronic kidney disease; PAD, peripheral artery disease; HF, heart failure. | | | | | |

| **Supplementary Table S2. Hazard ratios and 95% confidence intervals for the incidence of** **stroke, myocardial infarction, and all-cause mortality during follow up by quartiles of baseline gamma-glutamyl transferase ^a^** | | | | | | | | |
| --- | --- | --- | --- | --- | --- | --- | --- | --- |
| Baseline GGT quartiles | Events (n) | Follow-up  duration  (person-years) | Incidence rate  (per 1000  person-years) | Unadjusted  HR (95% CI) | Multivariate-adjusted  HR (95% CI) | | |  |
|  |  |  |  |  | Model 1 |  | Model 2 |  |
| **Stroke** | |  |  |  |  | |  | |
| Q1 (n=171673) | 6749 | 1051865.8 | 6.42 | 1(Ref.) | 1(Ref.) | | 1(Ref.) | |
| Q2 (n=185682) | 7164 | 1146792.6 | 6.25 | 0.97 (0.94‒1.01) | 1.11 (1.07‒1.14) | | 1.11 (1.08‒1.15) | |
| Q3 (n=168109) | 6158 | 1044799.6 | 5.89 | 0.92 (0.89‒0.95) | 1.19 (1.15‒1.23) | | 1.21 (1.16‒1.25) | |
| Q4 (n=173473) | 6048 | 1074868.6 | 5.63 | 0.88 (0.85‒0.91) | 1.32 (1.27‒1.37) | | 1.34 (1.29‒1.40) | |
| **Myocardial infarction** | |  |  |  |  | |  | |
| Q1 (n=171673) | 3898 | 1059777.9 | 3.68 | 1(Ref.) | 1(Ref.) | | 1(Ref.) | |
| Q2 (n=185682) | 4330 | 1155009.4 | 3.75 | 1.02 (0.98‒1.06) | 1.13 (1.08‒1.18) | | 1.15 (1.10‒1.20) | |
| Q3 (n=168109) | 3618 | 1052397.2 | 3.44 | 0.93 (0.89‒0.98) | 1.14 (1.09‒1.20) | | 1.17 (1.12‒1.23) | |
| Q4 (n=173473) | 3257 | 1082692.8 | 3.01 | 0.82 (0.78‒0.86) | 1.13 (1.08‒1.19) | | 1.17 (1.11‒1.23) | |
| **All-cause mortality** | |  |  |  |  | |  | |
| Q1 (n=171673) | 12046 | 1069491.6 | 11.26 | 1(Ref.) | 1(Ref.) | | 1(Ref.) | |
| Q2 (n=185682) | 10428 | 1166370.9 | 8.94 | 0.79 (0.77‒0.81) | 1.11 (1.08‒1.14) | | 1.11 (1.08‒1.14) | |
| Q3 (n=168109) | 8420 | 1062076.8 | 7.93 | 0.70 (0.68‒0.72) | 1.24 (1.21‒1.28) | | 1.24 (1.20‒1.28) | |
| Q4 (n=173473) | 9088 | 1091649.2 | 8.33 | 0.74 (0.72‒0.76) | 1.67 (1.62‒1.72) | | 1.65 (1.60‒1.70) | |

^a^ Q1: 4–25 (men), 4–16 (women) U/L; Q2: 26–38 (men), 17–23 (women) U/L; Q3: 39– 63 (men), 24–33 (women) U/L; Q4: 64–1000 (men), 34– 1000 (women) U/L.

Model 1 is adjusted for age, sex, baseline estimated glomerular filtration rate, body mass index, moderate drinking, current smoking, regular exercise, presence of hypertension and dyslipidemia, hemoglobin, and lowest 20% income. Model 2 is the same as model 1, plus adjustment for glucose, duration of diabetes, number of prescriptions for oral anti-diabetic medication, prescriptions for insulin, and presence of diabetic retinopathy and peripheral artery disease.

**Supplementary Table S3. Hazard ratios and 95% confidence intervals for the incidence of stroke, myocardial infarction, and all-cause mortality during follow up by quartiles of gamma-glutamyl transpeptidase variability assessed by average successive variability in each quartile of baseline gamma-glutamyl transpeptidase**

| Baseline  GGT  quartile | GGT  ASV  quartile | Stroke | | | | Myocardial infarction | | | | All-cause mortality | | | |
| --- | --- | --- | --- | --- | --- | --- | --- | --- | --- | --- | --- | --- | --- |
|  |  | Duration  (person-years) | Events  (n) | Incidence rate  (per 1000  person-years) | Multivariate-adjusted HR  (95% CI) ^*^ | Duration  (person-years) | Events  (n) | Incidence rate  (per 1000  person-years) | Multivariate-adjusted HR  (95% CI) ^*^ | Duration  (person-years) | Events  (n) | Incidence rate  (per 1000  person-years) | Multivariate-adjusted HR  (95% CI) ^*^ |
| Q1 | Q1 (N=51036) | 314203.3 | 1851 | 5.89 | 1(Ref.) | 316348.0 | 1081 | 3.42 | 1(Ref.) | 319103.4 | 3254 | 10.20 | 1(Ref.) |
|  | Q2 (N=44457) | 274602.1 | 1677 | 6.11 | 1.02 (0.96‒1.09) | 276607.4 | 975 | 3.52 | 1.02 (0.93‒1.11) | 278980.6 | 2903 | 10.41 | 1.02 (0.97‒1.07) |
|  | Q3 (N=40771) | 249353.8 | 1652 | 6.63 | 1.08 (1.01‒1.16) | 251266.5 | 972 | 3.87 | 1.09 (0.99‒1.19) | 253577.9 | 2959 | 11.67 | 1.13(1.07‒1.18) |
|  | Q4 (N=35409) | 213706.6 | 1569 | 7.34 | 1.15 (1.07‒1.23) | 215556.1 | 870 | 4.04 | 1.09 (0.99‒1.19) | 217829.7 | 2930 | 13.45 | 1.22(1.16‒1.29) |
| Q2 | Q1 (N=55590) | 343882.7 | 2092 | 6.08 | 1(Ref.) | 346415.9 | 1246 | 3.60 | 1(Ref.) | 349639.8 | 2852 | 8.16 | 1(Ref.) |
|  | Q2 (N=50498) | 314694.0 | 1924 | 6.11 | 1.04 (0.98‒1.10) | 316777.8 | 1186 | 3.74 | 1.06 (0.98‒1.15) | 319880.3 | 2586 | 8.08 | 1.04 (0.99‒1.10) |
|  | Q3 (N=43800) | 270646.7 | 1702 | 6.29 | 1.04 (0.98‒1.11) | 272411.1 | 1063 | 3.90 | 1.08 (0.99‒1.17) | 275343.2 | 2576 | 9.36 | 1.16 (1.10‒1.22) |
|  | Q4 (N=35794) | 217569.2 | 1446 | 6.65 | 1.05 (0.98‒1.13) | 219404.6 | 835 | 3.81 | 1.01 (0.93‒1.10) | 221507.6 | 2414 | 10.9 | 1.26 (1.20‒1.34) |
| Q3 | Q1 (N=41516) | 257710.8 | 1498 | 5.81 | 1(Ref.) | 259694.5 | 865 | 3.33 | 1(Ref.) | 261983.8 | 1852 | 7.07 | 1(Ref.) |
|  | Q2 (N=44135) | 277410.1 | 1549 | 5.58 | 0.99 (0.93‒1.07) | 279076.2 | 971 | 3.48 | 1.07 (0.97‒1.17) | 281740.7 | 1949 | 6.92 | 1.02 (0.95‒1.08) |
|  | Q3 (N=43628) | 272133.1 | 1562 | 5.74 | 1.01 (0.94‒1.08) | 274043.9 | 930 | 3.39 | 1.02 (0.93‒1.12) | 276556.2 | 2214 | 8.01 | 1.12 (1.05‒1.19) |
|  | Q4 (N=38830) | 237545.5 | 1549 | 6.52 | 1.08 (1.00‒1.16) | 239582.6 | 852 | 3.56 | 1.02 (0.92‒1.12) | 241796.0 | 2405 | 9.95 | 1.23 (1.16‒1.31) |
| Q4 | Q1 (N=26625) | 164433.3 | 925 | 5.63 | 1(Ref.) | 165720.2 | 481 | 2.90 | 1(Ref.) | 167054.3 | 1079 | 6.46 | 1(Ref.) |
|  | Q2 (N=35568) | 223484.2 | 1162 | 5.20 | 0.95 (0.87‒1.04) | 224945.1 | 631 | 2.81 | 0.98 (0.87‒1.11) | 226820.5 | 1449 | 6.39 | 1.01 (0.94‒1.10) |
|  | Q3 (N=46592) | 291970.3 | 1514 | 5.19 | 0.92 (0.85‒1.00) | 293804.2 | 877 | 2.98 | 1.02 (0.92‒1.14) | 296125.4 | 2142 | 7.23 | 1.09 (1.01‒1.17) |
|  | Q4 (N=64688) | 394980.7 | 2447 | 6.20 | 0.97 (0.90‒1.05) | 398223.3 | 1268 | 3.18 | 0.99 (0.89‒1.10) | 401649.0 | 4418 | 11.0 | 1.32 (1.24‒1.41) |

^*^ Adjusted for age, sex, baseline estimated glomerular filtration rate, body mass index, moderate drinking, current smoking, regular exercise, presence of hypertension and dyslipidemia, hemoglobin, lowest 20% income, glucose, duration of diabetes, number of prescriptions for oral anti-diabetic medication, prescriptions for insulin, and presence of diabetic retinopathy and peripheral artery disease.

**
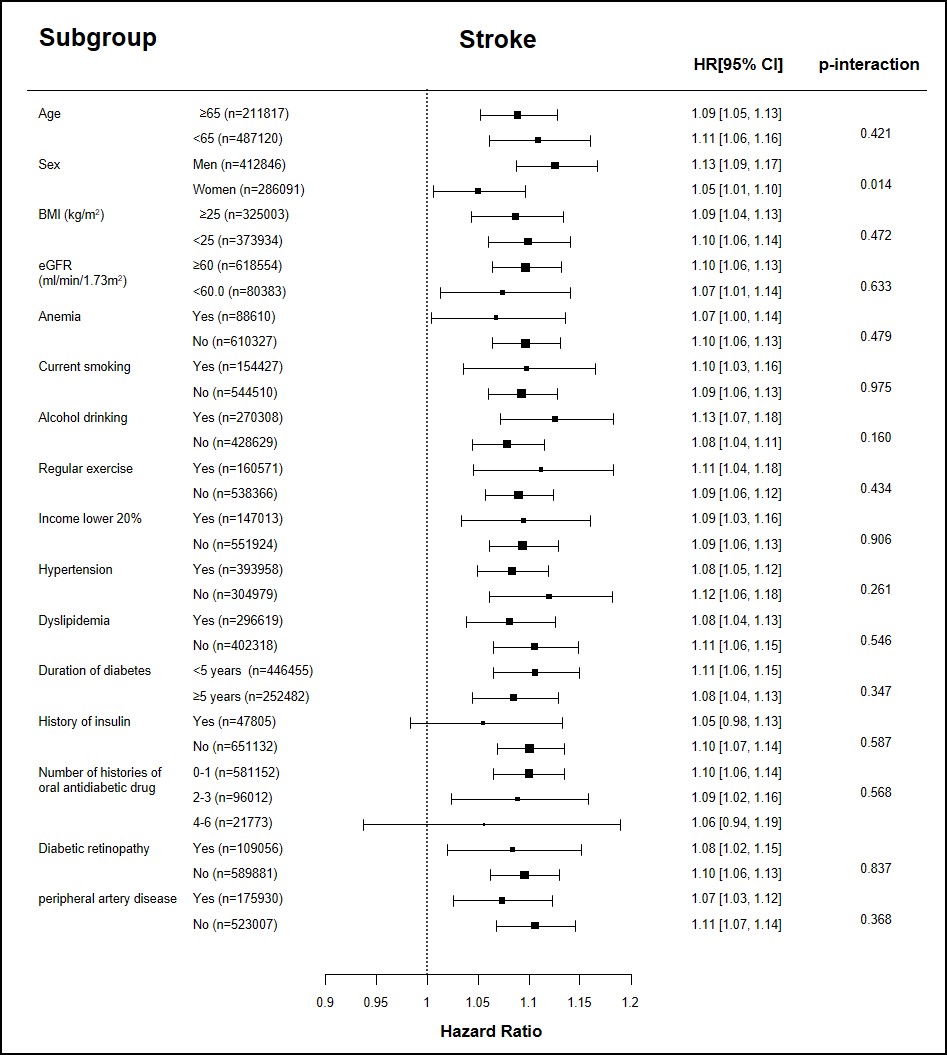
**

**Supplementary Figure S2.** Hazard ratios and 95% confidence intervals for the incidence of stroke in the highest quartile versus the other three quartiles of gamma-glutamyl transferase variability assessed by average successive variability in subgroup analyses adjusted for age, sex, baseline estimated glomerular filtration rate, body mass index, moderate drinking, current smoking, regular exercise, presence of hypertension and dyslipidemia, hemoglobin, lowest 20% income, glucose, duration of diabetes, number of prescriptions for oral anti-diabetic medication, prescriptions for insulin, and presence of diabetic retinopathy and peripheral artery disease.

**
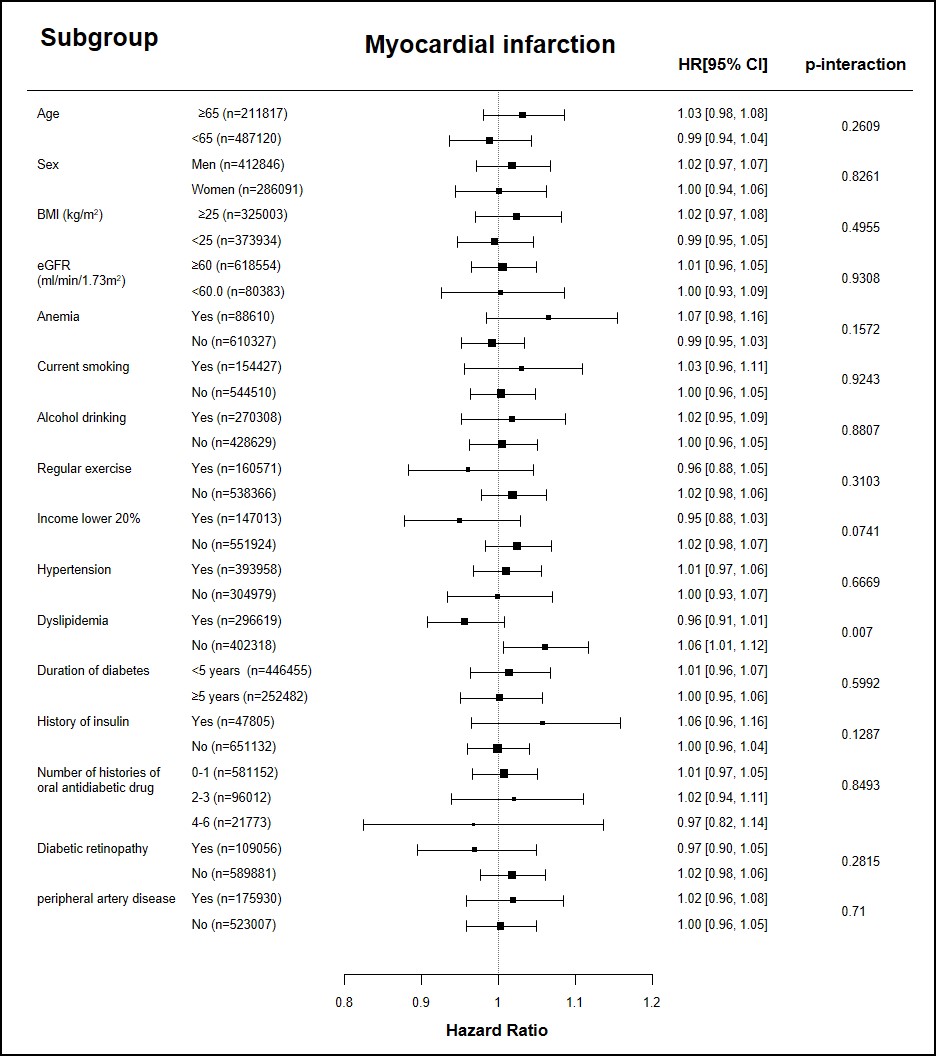
**

**Supplementary Figure S3.** Hazard ratios and 95% confidence intervals for the incidence of myocardial infarction in the highest quartile versus the other three quartiles of gamma-glutamyl transferase variability assessed by average successive variability in subgroup analyses adjusted for age, sex, baseline estimated glomerular filtration rate, body mass index, moderate drinking, current smoking, regular exercise, presence of hypertension and dyslipidemia, hemoglobin, lowest 20% income, glucose, duration of diabetes, number of prescriptions for oral anti-diabetic medication, prescriptions for insulin, and presence of diabetic retinopathy and peripheral artery disease.
